# Sick Headache

**Published:** 1883-07

**Authors:** 


### SICK HEADACHE

IS sickness at stomach, a tendency to vomit, combined with pain in some part of the head, generally the left side. It is caused by there being too much bile in the system, from the fact that this bile is manufactured too rapidly, or is not worked out of the system fast enough by steady, active exercise. Hence, sedentary persons—those who do not walk about a great deal, but are seated in the house nearly all the time—are almost exclusively the victims of this distressing malady. It usually begins soon after waking up in the morning, and lasts a day or two or more. There are many causes; the most frequent is derangement of the stomach by late and hearty suppers; by eating too soon after a regular meal (five hours should, at least, intervene); eating without an appetite; forcing food; eating after one is conscious of having had enough; eating too much of any favorite dish; eating something which the stomach cannot digest, or sour stomach. Any of these things may induce sick headache; all of them can be avoided.

Over-fatigue or great mental emotion of any kind, a severe mental application, have brought on sick headache of the most distressing character in an hour; it is caused by indulgence in spirituous liquors. Decayed teeth are a frequent cause of the severest form of sick headache through irritation to the nerves, and blood-poisoning from the ulcerated teeth.

When a person has sick headache there is no appetite ; the very sight of food is hateful ; the tongue is furred ; the feet and hands are cold, and there is a feeling of general discomfort, with an utter indisposition to do anything whatever.

A glass of warm water, into which has been rapidly stirred a heaping teaspoonful each of salt and mustard, by causing instantaneous vomiting, empties the stomach of the bile or indigested sour food, and a grateful relief is often experienced at once ; and rest, with a few hours of sound, refreshing sleep, completes the cure, especially if the principal part of the next day or two is spent in mental diversion and out-door activities, not eating an atom of food —but drinking freely of cold water or hot teas—until you feel as if a piece of plain, cold bread and butter would "taste really good." Nine times in ten the cause of sick headache is in the fact that the stomach was not able to digest the food last introduced into it, either from its having been unsuitable, or excessive in quantity.

When the stomach is weak, a spoonful of the mildest, blandest food would cause an attack of sick headache, when ten times the amount might be taken in health, not only with impunity, but with positive advantage.

Those who are subject to "sick headache" eat too much and exercise too little, and have cold feet and constipation. There is also, in a majority of cases, neuralgia of the face caused by decayed teeth, or ulceration at the roots of teeth that appear to be sound. When this is the case there will be no permanent relief, while life lasts, without the assistance of a competent dentist. But where the teeth are not at fault, a diet of cold bread and butter, and ripe fruit or berries, with moderate, continuous exercise in the open air sufficient to keep up a very gentle perspiration, would of themselves cure almost any case within thirty-six hours. Two teaspoonfuls of pulverized charcoal, stirred in half a glass of water and drank, generally gives prompt relief.

